# SARS-CoV-2-specific humoral and cellular immune responses to BNT162b2 vaccine in Fibrodysplasia ossificans progressiva patients

**DOI:** 10.3389/fimmu.2022.1017232

**Published:** 2022-11-09

**Authors:** Jitka Smetanova, Tomas Milota, Michal Rataj, Jana Hurnakova, Hana Zelena, Rudolf Horvath

**Affiliations:** ^1^ Department of Immunology, Second Faculty of Medicine Charles University and Motol University Hospital, Prague, Czechia; ^2^ Department of Paediatric and Adult Rheumatology, Motol University Hospital, Prague, Czechia; ^3^ Department of Virology, Public Health Institute, Ostrava, Czechia

**Keywords:** fibrodysplasia ossificans progressive, COVID-19, SARS-CoV-2, vaccination, immunogenicity, safety

## Abstract

**Introduction:**

Fibrodysplasia ossificans progressiva (FOP) is characterized by progressive heterotopic ossification triggered by various conditions, such as trauma, infection, including COVID-19 infection, and vaccination. Although SARS-CoV-2 vaccinations prevent poor outcomes in the general population, there is limited evidence on safety, immunogenicity, and efficacy of SARS-CoV-2 vaccines for inpatients with FOP.

**Methods:**

A case series of two patients with FOP focused on humoral, cellular post-vaccination response, and the incidence of adverse events after administration of the BNT162b2 vaccine (Comirnaty).

**Results:**

Injection site reactions, fever, myalgia, and fatigue were the most common adverse events (AE). Neither severe AE (SAE), nor disease flare-ups were observed. No differences between patients with FOP and healthy controls were observed in humoral and cellular responses.

**Conclusions:**

The BNT162b2 vaccine induced high humoral and cellular response levels in patients with FOP. Vaccination was not associated with SAE or disease relapse. The AEs spectrum was comparable to that of the general population.

## Introduction

COVID-19, caused by the severe acute respiratory syndrome coronavirus 2 (SARS-CoV-2), was first identified in the Chinese city of Wuhan in December 2019 (1). Since then, the disease has spread rapidly worldwide and was announced as a global pandemic in March 2020. COVID-19 has become a significant concern, with more than four-hundred million infected individuals and almost six million confirmed deaths recorded by March 2022 (2). Numerous reports indicated that COVID-19-associated complications and more severe clinical outcomes occurred in patients with risk factors such as higher age (> 75 years), severe obesity, male sex, and chronic cardiovascular and pulmonary conditions (3, 4). However, only limited evidence is available for rare diseases.

Fibrodysplasia ossificans progressiva (FOP) is an ultra-rare genetic condition caused by mutations in the *ACVR1/ALK2* gene that affects approximately 1 in 2 million people worldwide (5). The disease is characterized by the development of progressive heterotopic ossification (HO) of soft tissues that span joints, resulting in an ectopic skeleton. Consequently, the progressive nature of the disease usually leads to severe patient disability (6, 7). Various devastating complications can occur, including thoracic insufficiency (8). HO formations can be triggered for no specific reason; however, some are linked to inflammation arising from various tissue injuries. These can be caused by trauma, interventional medical procedures or intramuscular immunizations among others (9, 10). Therefore, patients with FOP are anticipated to be at high risk of adverse outcomes related to COVID-19. In addition, COVID-19 alone may serve as a possible disease-flare trigger, as recently described (11, 12). Thus, disease prevention should be paramount for patients with FOP.

Although preventive measures, in general, can provide satisfactory short-term results; in the long-term they are unsustainable and mostly fail, particularly due to significant negative socio-economic impacts (13, 14). Such negative effects have also been reported in FOP (15). Thus, in the absence of causative treatment, vaccination against COVID-19 might be an essential strategy to prevent rapid spread of the disease and alleviate disease severity in infected individuals. Across all approved vaccines, in the general population, their high efficacy, good safety profile, and high degree of immunogenicity have been demonstrated in both clinical trials (16–20) and real-world application (21–23). Nevertheless, only very limited data on the efficacy and safety in specific and vulnerable populations such as FOP currently exists (24).

In our study, we aimed to investigate both humoral and cellular immune responses in FOP patients after two doses of the BNT162b2 vaccination. Our goal was to describe the specific elicited humoral and cellular immunity response against SARS-CoV-2. We also tested vaccine safety regarding injection site reactions and disease flare-ups following the vaccination procedure.

## Methods

### Study design

**Figure 1:**
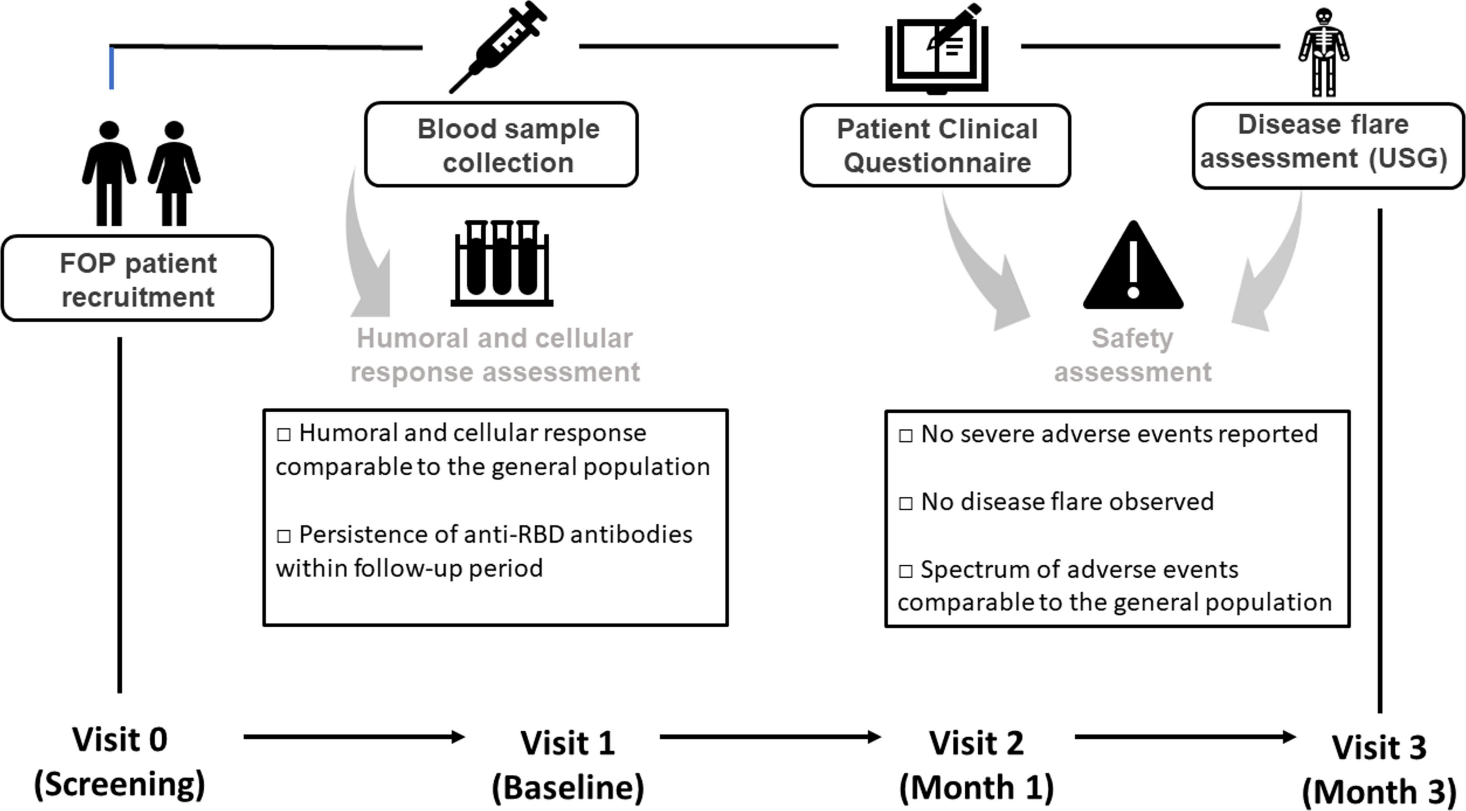
Timeline of the scheduled visits after vaccination. Immunization was performed according to International Clinical Council on Fibrodysplasia ossificans progressiva (ICC on FOP) Guidelines (27).

The study was designed as a prospective observational clinical trial evaluating the cellular and humoral immunogenicity of the mRNA vaccine BNT162b (Comirnaty). The study was conducted between November 2021 to January 2022. All study protocols were carried out in accordance with the Ethical Standards of the Institutional Research Committee - the Ethics Committee of the University Hospital Motol in Prague (Ref.no. EK-753.1.3/21 approved on 10 June 2021). The observation followed the Strengthening the Reporting of Observational Studies in Epidemiology (STROBE) recommendations (25). Blood samples, clinical and safety data were collected at four study visits: **Visit 0 (screening)** – verification of the FOP diagnosis, indication of vaccination, vaccination according to ICC on FOP; **Visit 1 (baseline)** – patient’s recruitment (evaluation of the inclusion/exclusion criteria) and disease activity assessment (clinical evaluation) (26); **Visit 2 (follow-up, month 1)** – disease activity assessment, safety data collection (patient clinical questionnaire, PCQ) and blood sample collection (total blood count with differential, biochemical and immunological parameters including inflammatory markers, humoral and T cell post-vaccination response assessment); and **Visit 3 (follow-up, month 3)** - disease activity assessment (clinical evaluation, deltoid muscle ultrasonography), blood sample collection (humoral post-vaccination response). The study design is summarized in **Figure 1**. The blood samples were collected in EDTA (T cell response, total blood count with differential) and clot activator tubers (humoral response, biochemical and immunological parameters).

### Study population

All patients were enrolled upon assessment of inclusion: 1. genetically confirmed diagnosis and/or corresponding clinical and radiographic features; 2. vaccination indicated by attending rheumatologist and performed in specialized vaccination centers according to the International Clinical Council (ICC) on FOP guidelines (27); and 3. signed patient informed consent, and exclusion criteria: 1. previous exposure to COVID-19 infection based on RT-PCR positivity or the presence of IgG anti-SARS-CoV-2 specific antibodies; and 2. an acute respiratory tract infection four weeks prior to vaccination. The FOP cohort was compared to the corresponding sex- and age-matched healthy control (HC) group.

All included patients were administered two 0.3ml (30 ug of mRNA) standard doses of the BNT162b2 vaccine intramuscularly into the deltoid muscle at three week intervals as recommended by the SPC prior to Visit - 1. Vaccination with the mRNA BNT162b2 vaccine Comirnaty was performed in government-authorized specialized vaccination centers.

### Humoral response assessment

Anti-spike 1 SARS-CoV-2 IgG titers (positive cut-off value >18 U/ml) were measured using ELISA COVID-19 RBD IgG (Test-Line Clinical Diagnostics, Brno, Czechia); anti- nucleocapsid (NCP), anti-spike 2, and receptor-binding domain (RBD) autoantibodies (positive cut-off value >180 U/ml) by immunoblot assay (Microblot-Array COVID-19 IgG, Test-Line Clinical Diagnostics).

The virus neutralization test (VNT) was performed according to previously published protocol using the SARS-CoV-2 strain extracted from a clinical sample (hCoV-19/Czech Republic/NRL_9640/2020|EPI_ISL_626593) and CV-1 cells (African green monkey kidney fibroblasts). Serum samples were diluted in two-fold dilutions which, after mixing with the virus, resulted in a final serum concentration of 1/10 up to 1/560. These dilutions were mixed with the virus solution (100 infection doses) and incubated. Then, 25 µL of neutral red dye (1:10,000 aqueous solution) was added. The uninfected results were red at the end of incubation. Only live uninfected cells were stained with the neutral red dye, enabling a macroscopic reading, and deeming the use of a microscope unnecessary. The results of the VNT were expressed in the form of virus neutralization titer, which represents an inverted value of the highest dilution of the sample neutralizing the cytopathic effect of the virus for more than 50% of the cells. The titer was calculated from the volume of serum and virus at the endpoint of the median tissue culture infectious dose (TCID_50_). Positivity was determined by a titer of 20 and above (28).

### Stimulation assays

Stimulation with anti-human CD3 purified low endotoxin (0.29 ug/ml) served as an (unspecific) positive control, PepMix™ (0.1 µg/µl) SARS-CoV-2 as specific response (JPT Peptide Technologies GmbH, Berlin, Germany) and with the costimulatory antibody, CD28/CD49d (100 mg/ml). Subsequently, Brefeldin A (0.01 mg/ul, Sigma Aldrich, St. Luis, USA) was added to the samples, and the samples were incubated for another 4 hours at 37°C. Stimulation assays followed previously published protocols (29, 30).

### Flow cytometry

After incubation, the PBMCs were washed and stained by fluorescent conjugated monoclonal antibodies for fluorescence-activated cell sorting analysis (FACS antibodies) against CD3 and CD4. After fixation and permeabilization with the permeabilization buffer 10x, eBioscience™ fixation/perm diluent, and fixation/permeabilization concentrate (Thermo Fisher Scientific), the PBMCs were further stained with anti-IFNγ and anti-TNFα FACS antibodies (characteristics of FACS antibodies in **Supplementary Table 1**). Finally, samples were measured on the flow cytometer FACS Fortessa (BD Biosciences, San Diego, USA), and data were analyzed using the FlowJo software (version 10.6.1, BD Biosciences). Gating strategy is shown in **Supplementary Figures 1A-C**. The percentage of TNFalpha+CD4+ and INFgamma+CD4+ cells from CD4+ cells (all) and response ratio (RR, the proportion of stimulated and unstimulated cells) were calculated.

### Safety and quality of life assessment

AEs were reported using PCQ focused on local (injection site reactions) and systemic reactions (fever, headache, myalgia, arthralgia), and emergency medication (such as analgesic/antipyretics drugs). The intensity of pain was assessed on a 10-point visual analog scale (VAS). SAEs were defined as an acute condition requiring hospital admission or urgent medical intervention following the vaccination. Late local adverse reactions were assessed by deltoid muscle ultrasonography (USG) focusing on the detection of HO (31). US examinations were performed using MyLab ClassC (Esaote S.p.A. Genoa, Italy), equipped with a linear probe with a frequency ranging from 10–18 MHz.

The spectrum of routine laboratory parameters - total blood count and differential, biochemistry (ALT, AST, GGT, ALP, urea, creatinine), immunology (antinuclear autoantibodies, anti-neutrophilic autoantibodies, IgM/IgA/IgM rheumatoid factors), and inflammatory markers (C-reactive protein) were assessed. Quality of life was assessed using EQ-5D (32) and a health assessment questionnaire (HAQ) (33).

## Results

A thirty-one-year-old male and a twenty-nine-year-old female with FOP were recruited. Both patients had late-stage disease with severe disability and impaired quality of life: HAQ (2.875) and EQ-5D (-0.166), and HAQ (2.375) and EQ-5D (0.587), respectively. The male patient also developed significant comorbidities, including obesity, type 1 diabetes, and psoriasis. He tested positive for SARS-CoV-2 by RT-PCR after epidemiologically significant contact with a COVID-19 positive person within the follow-up period. The patient did not develop any COVID-19 symptoms and did not require hospital admission, oxygen therapy or specific treatment, such as anti-SARS-CoV-2 monoclonal antibodies or antiviral drugs. In contrast, the female patient had no other significant comorbidity and did not test positive for COVID-19.

### Safety analysis

The male patient reported localized pain at the injection site (VAS 2/10) after the first and second vaccine doses. This patient also had generalized myalgia (VAS 2/10). None of the AE required emergency medications. The female patient reported very intense pain at the injection site (VAS 9/10) accompanied by a localized, self-limited itchy rash. This patient also had a short-term fever following both vaccine doses that required emergency medication (ibuprofen). In addition, the patient also reported fatigue. After the three month follow-up period, neither clinical signs of disease flare nor any new HO formation at the injection site was detected using US examination in either patient (**Supplementary Figures 2A, B**). Neither patient experienced an SAE.

Following vaccination, no induced autoantibodies (antinuclear autoantibodies, anti-neutrophilic autoantibodies, and IgM/IgA/IgM rheumatoid factors). No significant changes in total blood count or differential biochemical parameters (ALT, AST, GGT, ALP, urea, creatinine and high-sensitive CRP) were observed within the three month follow-up period (**Supplementary Table 2**).

### Specific humoral responses

**Figure 2:**
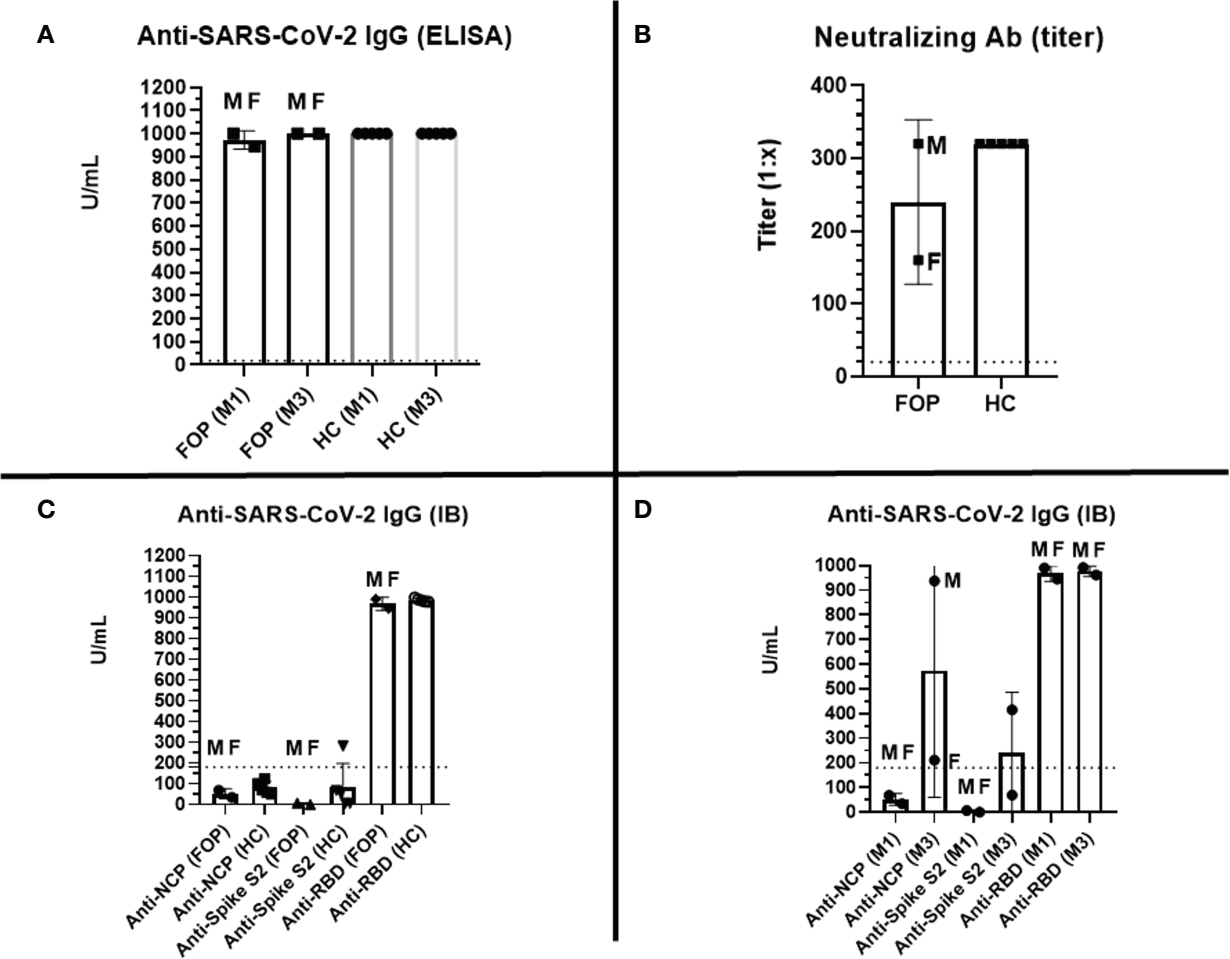
Specific post-vaccination humoral response –level (U/ml, positive cut-off value > 18 U/ml, marked as dashed line) of specific anti-RBD SARS-CoV-2 IgG antibodies measured by ELISA at months 1 (M1) and 3 (M3) in healthy controls (HC) and patients with Fibrodysplasia ossificans progressiva (FOP, M- male patient #1, F- female patient #2) **(A)**; titer (1:X) of specific anti-SARS-CoV-2 neutralizing antibodies at M1 in HC and FOP **(B)**; level (U/ml, positive cut-off value > 180 U/ml, marked as dashed line) of specific anti- SARS-CoV-2 specific IgG antibodies (spike S1; spike S2; NCP, nucleocapsid protein; RBD, receptor-binding domain) measured by immunoblot (IB) at M1 **(C)**, and M3 **(D)** in HC and FOP.

Strong anti-spike-1 SARS-CoV-2 responses were detected using ELISA in both FOP patients following vaccination. Specific antibody concentrations were comparable to healthy controls (HC) (mean 972.1 U/ml vs. 1,000 U/ml) (**Figure 2A**). The humoral post-vaccination response was confirmed using western blot assay in both groups: mean concentration of anti-RBD SARS-CoV-2 antibodies was 967.8 U/ml (FOP patients) vs. 986.1 U/ml (HC) (**Figure 2C**). Other anti-SARS-CoV-2 antibodies (anti-NCP, anti-spike 2) were detected below the upper reference limit, suggesting no previous exposure to COVID-19 in either cohort in month 1 (**Figure 2D**). The high level of anti-RBD SARS-CoV-2 antibodies persisted as measured by ELISA (mean 1,000 U/ml) and WB (976.8 U/ml) in the third month (**Figures 2A, D**). However, the high titers of anti-NCP and anti-spike 2 specific antibodies were detected in patient 1, reflecting a convalescent state (**Figure 2D**). The detected anti-SARS-CoV-2 antibodies had virus-neutralizing activity in both FOP and HC individuals, with no differences in their titers (mean titer 1:240, range 1:160–1:320 vs. 1:320) (**Figure 2B**). A higher titer of neutralizing antibodies was identified in the patient after COVID-19 recovery.

### Cellular responses

**Figure 3:**
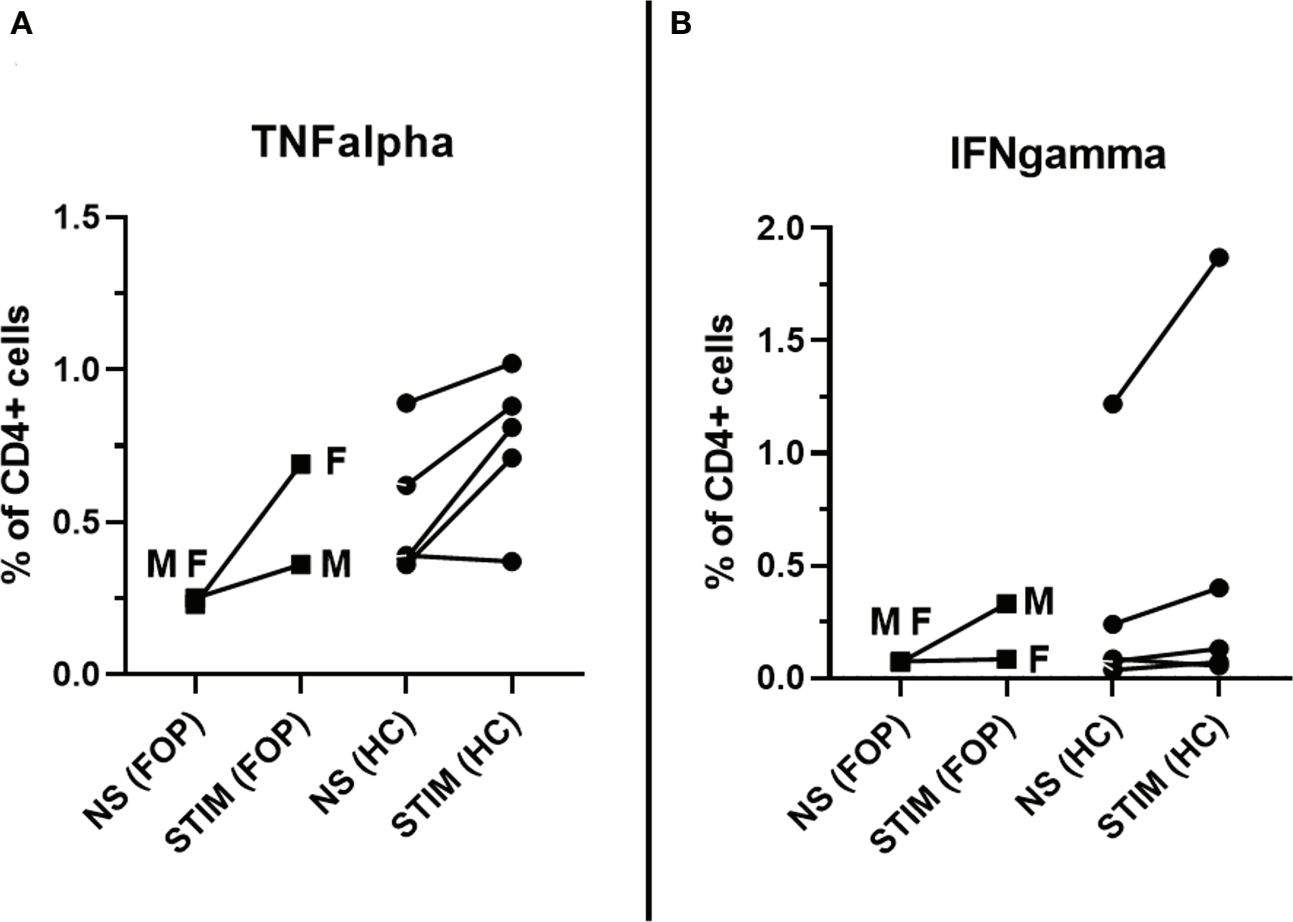
Specific post-vaccination cellular response – measured as percentage (%) of CD4+ T cells producing TNFα **(A)** or IFN-γ **(B)** in unstimulated cells (NS) and after stimulation (STIM) at month 1 in healthy controls (HC) and patients with Fibrodysplasia ossificans (FOP, M- male patient #1, F- female patient #2).

After specific stimulation, 0.53% and 0.76% of TNFα+CD4+ cells were detected in FOP patients and HC, respectively, corresponding to 2.2 and 1.52 RR. RR >1.5 observed in 1/2 (50%) FOP patients and 2/5 (40%) HC patients. Similar results were obtained when IFN-γ +CD4+ cells were measured; 0.21% and 0.5% of IFN-γ +CD4+ cells were identified in FOP and HC individuals, respectively. This corresponded to 2.96 and of 1.51 RR. RR >1.5 seen in 1/2 (50%) of FOP patients and 4/5 (80%) in the HC. A higher IFN-γ response was detected in patients after SARS-CoV-2 infection (male patient) (**Figures 3A, B**).

## Discussion

Patients with FOP are considered vulnerable to COVID-19 and COVID-19-related events owing to pre-existing lung impairment or higher risk of FOP flares and HO formation that may be linked to COVID-19 related intensive care management. It is worth mentioning that a viral infection itself, including but not limited to SARS-CoV-2, may lead to disease flare-ups (34). The risk of FOP flare-ups and HO in COVID-19 has been described in two case reports (11, 12). In addition, a small registry-based trial of the International FOP Association (IFOPA) Registry reported that two out of ten patients with confirmed SARS-CoV-2 infection experienced disease flare-ups. This observation also identified a significant social impact with decreased participation in the social activities of the patients during the pandemic (15). According to the ICC on FOP, precautionary measures remain a cornerstone for preventing COVID-19. However, the COVID-19 vaccination is a component of the updated ICC on FOP recommendations. The decision to vaccinate is made only after reviewing individual case files and carefully balancing risks and benefits. Despite this, intramuscular injections including vaccination may be associated with a higher risk for disease flare-ups and HO formation (35, 36), therefore, it is recommended to use the approved administration route, which is supported by results of clinical trials that led to authorization of the product (27). The majority of currently available anti-SARS-CoV-2 vaccines have been approved for intramuscular application. Any other method of administration may lead to a weak immune response or reduced tolerability (37). Nevertheless, data on efficacy, immunogenicity and safety to fully understand the role of anti-COVID-19 vaccination in FOP are still limited. Therefore, we initiated a prospective observation focusing on the safety and immunogenicity of the mRNA vaccine BNT162b2 in two patients.

Overall, the vaccine was well-tolerated with minor AEs (local pain at the injection site and limited pruritic rash, myalgia, fever, and fatigue) lasting for a couple of days. No SAEs were recorded. In addition, no clinical or USG signs of disease flare-ups or HO were documented within three months of vaccination. These findings are consistent with previously published observations by Kou et al. (2021) (15). Further, no significant changes were observed in hematological, biochemical, immunological, and inflammatory parameters within the three months following vaccination.

Next, we assessed the induction of specific humoral and cellular immune responses following vaccination. Our results suggest that vaccinated FOP patients could induce strong specific humoral responses against COVID-19, which does not differ from healthy individuals in magnitude and quality. Thus, anti-SARS-CoV-2 specific humoral responses appear to be unaltered in vaccinated FOP patients. Moreover, the three month follow-up period confirmed that the anti-RBD-specific antibody titers had sustained concentrations in both patients, comparable to those of HC. However, other antibodies (anti-NCAP and anti-spike 2) were detected in the male patient reflecting a convalescent state. A possible positive effect of vaccination was confirmed in the male patient. Despite late-stage disease and several risk factors, such as T1D and obesity, the male patient developed a PCR-confirmed asymptomatic COVID-19 infection. This observation is consistent with previous reports showing a decrease in the proportion of hospital admissions, complicated courses, and morbidity in vaccinated populations (38, 39).

The final part of our assessment focused on cellular immune responses following anti-COVID-19 vaccination. Several reports have demonstrated a significant proportion of SARS-CoV-2-specific T cell response in COVID-19 patients with various clinical symptoms, and individuals after vaccination with different anti-SARS-CoV-2 vaccines. This evidence suggests the important role of cellular immunity in anti-SARS-CoV-2 responses (40, 41). In our patients, we detected varying SARS-CoV-2 specific CD4+ responses. No significant differences were observed between the FOP and HC groups. However, the interpretation of vaccine-induced cellular immunity may be more complex; thus, more data is required. Our study had several limitations. First, the low number of included subjects does not allow any generalization of our results; second, we presumed that the magnitude of the cellular and humoral immune responses may change over time, thus, an extended follow-up period is required; and third, immunogenicity only provides a partial view of vaccine efficacy that should be confirmed by “real-life” evidence. However, any published data will be of interest to further our understanding and support ICC recommendations for safe, well-tolerated anti-SARS-CoV-2 vaccinations in FOP patients, especially to prevent the induction of significant humoral and cellular responses.

Additionally, several issues need to be addressed regarding booster doses. A predominant SASR-CoV-2 variant may influence the booster vaccine efficacy. While original mRNA anti-SARS-CoV-2 vaccines are less effective in the prevention of infection by the Omicron (42), a booster vaccination may reduce severe outcomes, including hospital admission in the general population (43) as well as immunocompromised (44) and other vulnerable populations (45). New generations of multivalent mRNA vaccines could provide better efficiency against novel variants along with an acceptable safety profile (46). High vaccination and infection frequencies also raise the question of hybrid immunity, which may be another critical factor in reducing infection risk (47). Further investigation is needed.

## Data availability statement

The raw data supporting the conclusions of this article will be made available by the authors, without undue reservation.

## Ethics statement

The studies involving human participants were reviewed and approved by Motol University Hospital Ethics Committee (EK-753.1.3/21; approved on 10-June-2021). The patients/participants provided their written informed consent to participate in this study. Written informed consent was obtained from the individual(s) for the publication of any potentially identifiable images or data included in this article.

## Author contributions

JS contributed to conceptualization; formal analysis; methodology; visualization; writing – original draft. TM contributed to conceptualization; data curation; formal analysis; funding acquisition; investigation; methodology; visualization; writing - original draft. MR contributed to investigation; methodology; validation. HZ contributed to investigation; methodology; validation. JH contributed to investigation; methodology; validation. RH contributed to conceptualization; data curation; investigation; methodology; supervision; validation; writing - review and editing. All authors contributed to the article and approved the submitted version.

## Funding

This project was supported by a grant from the Czech Health Research Council, Ministry of Health, Czech Republic (NU20-05-00320, NU22-05-00402) and the Ministry of Health, Czech Republic – Conceptual Development of Research Organization, Motol University Hospital, Prague, Czech Republic (00064203).

## Acknowledgments

The study was conducted in collaboration with rheumatologists from the Department of Pediatric and Adult Rheumatology, University Motol Hospital, Prague, Czech Republic who provided dispensary care for the participants, and with physicians from the Vaccination Center, University Motol Hospital, Prague, Czech Republic who administered the vaccines.

## Conflict of interest

The authors declare that the research was conducted in the absence of any commercial or financial relationships that could be construed as a potential conflict of interest.

## Publisher’s note

All claims expressed in this article are solely those of the authors and do not necessarily represent those of their affiliated organizations, or those of the publisher, the editors and the reviewers. Any product that may be evaluated in this article, or claim that may be made by its manufacturer, is not guaranteed or endorsed by the publisher.

## Supplementary material

Supplementary Figure 1
**(A)** Gating strategy showing lymphocytes gate (A), single cells gate (B), T cells (CD3+) gate (C), CD4+ and CD8+ cells gate (D), CD4+TNFα+ (E) and CD4+IFNγ+ cells gate (intracellular cytokine production, F), **(B)** CD4+TNFα+ and **(C)** CD4+IFNγ+ cells in negative control (unstimulated cells), positive control (CD3 and CD28/CD49d stimulation) and Stim-PEPMIX (specific stimulation with specific SARS-CoV-2 derived proteins and costimulation with CD28/CD49d).

Supplementary Figure 2 Ultrasound images of deltoid muscle performed at the month 3 follow-up visit. No new HO formations were detected at the injection site in both patients (**A**: male patient, H-Humerus, D-deltoid muscle with atrophy; **B**: female patient, H-Humerus, CA-anterior chief, CM-medium chief, CP-posterior chief of deltoid muscle).
