# Supplementary figures and images for: SARS-CoV-2-specific humoral and cellular immune responses to BNT162b2 vaccine in Fibrodysplasia ossificans progressiva patients
Source: Front Immunol. 2022 Nov 9;13:1017232. doi: 10.3389/fimmu.2022.1017232 (PMC9682080; doi:10.3389/fimmu.2022.1017232)

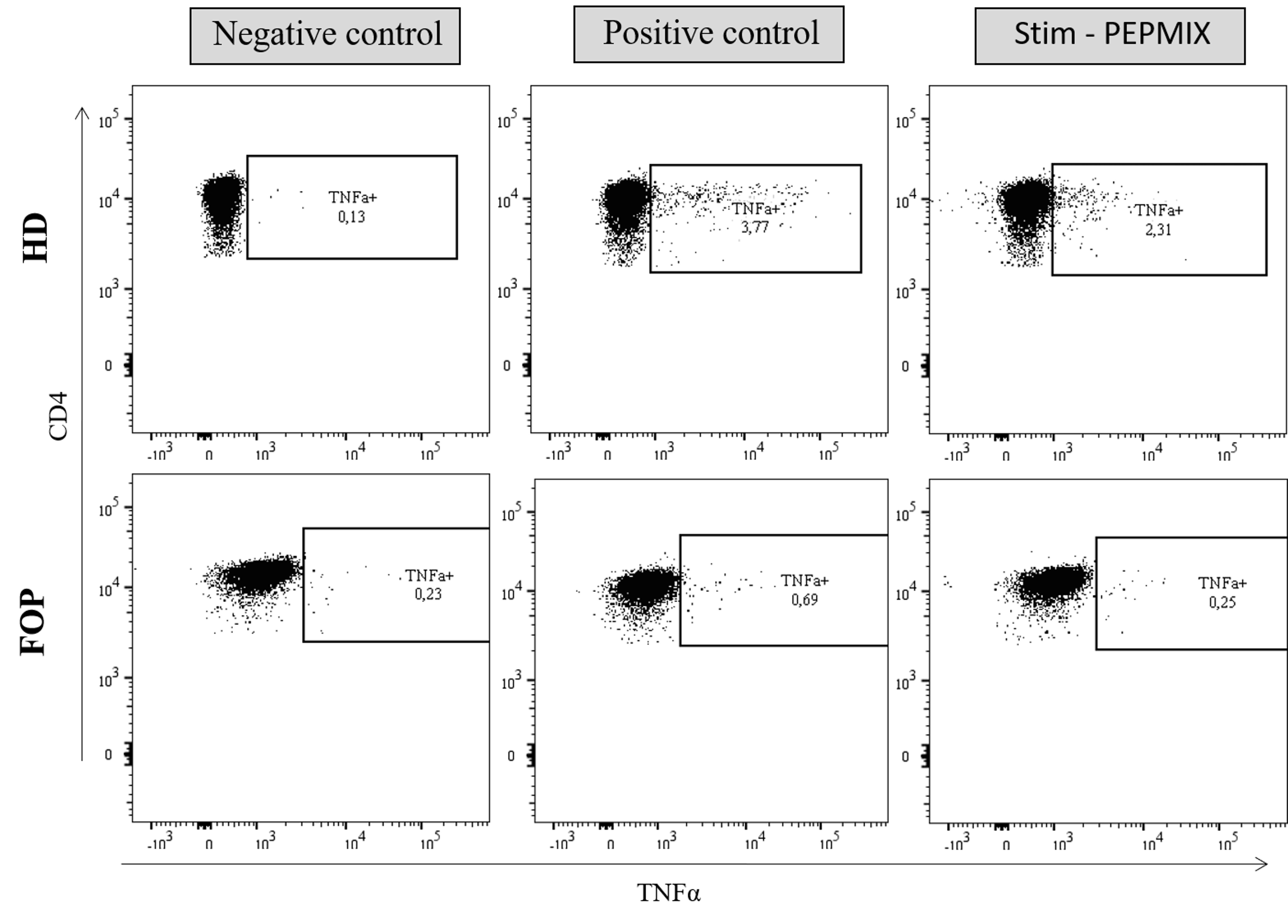

Supplement: Supplementary Figure 1 — (A) Gating strategy showing lymphocytes gate (A), single cells gate (B), T cells (CD3+) gate (C), CD4+ and CD8+ cells gate (D), CD4+TNFα+ (E) and CD4+IFNγ+ cells gate (intracellular cytokine production, F), (B) CD4+TNFα+ and (C) CD4+IFNγ+ cells in negative control (unstimulated cells), positive control (CD3 and CD28/CD49d stimulation) and Stim-PEPMIX (specific stimulation with specific SARS-CoV-2 derived proteins and costimulation with CD28/CD49d). [file Image_1.tif]

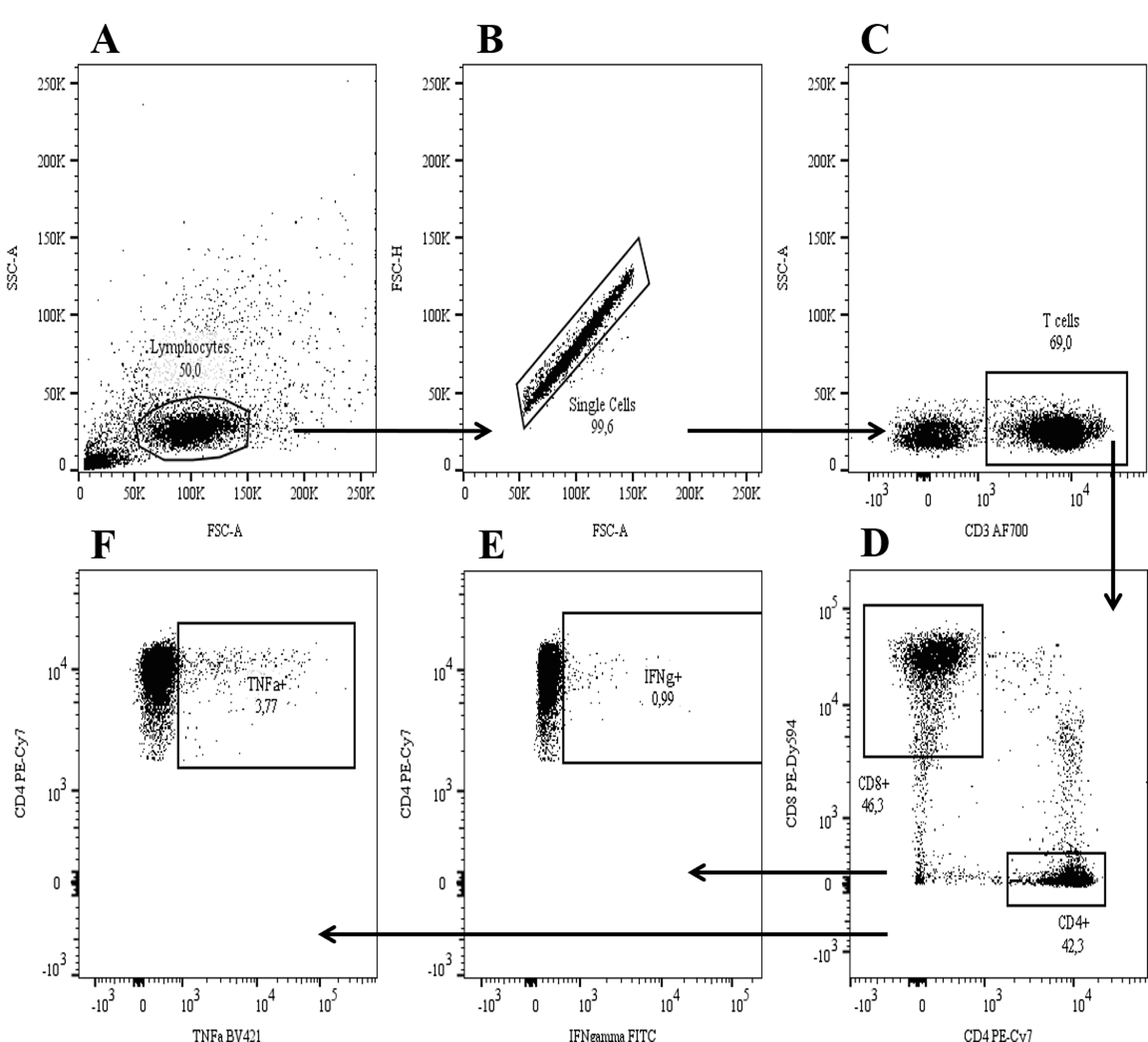

Supplement: Supplementary Figure 2 — Ultrasound images of deltoid muscle performed at the month 3 follow-up visit. No new HO formations were detected at the injection site in both patients (A: male patient, H-Humerus, D-deltoid muscle with atrophy; B: female patient, H-Humerus, CA-anterior chief, CM-medium chief, CP-posterior chief of deltoid muscle). [file Image_2.tif]

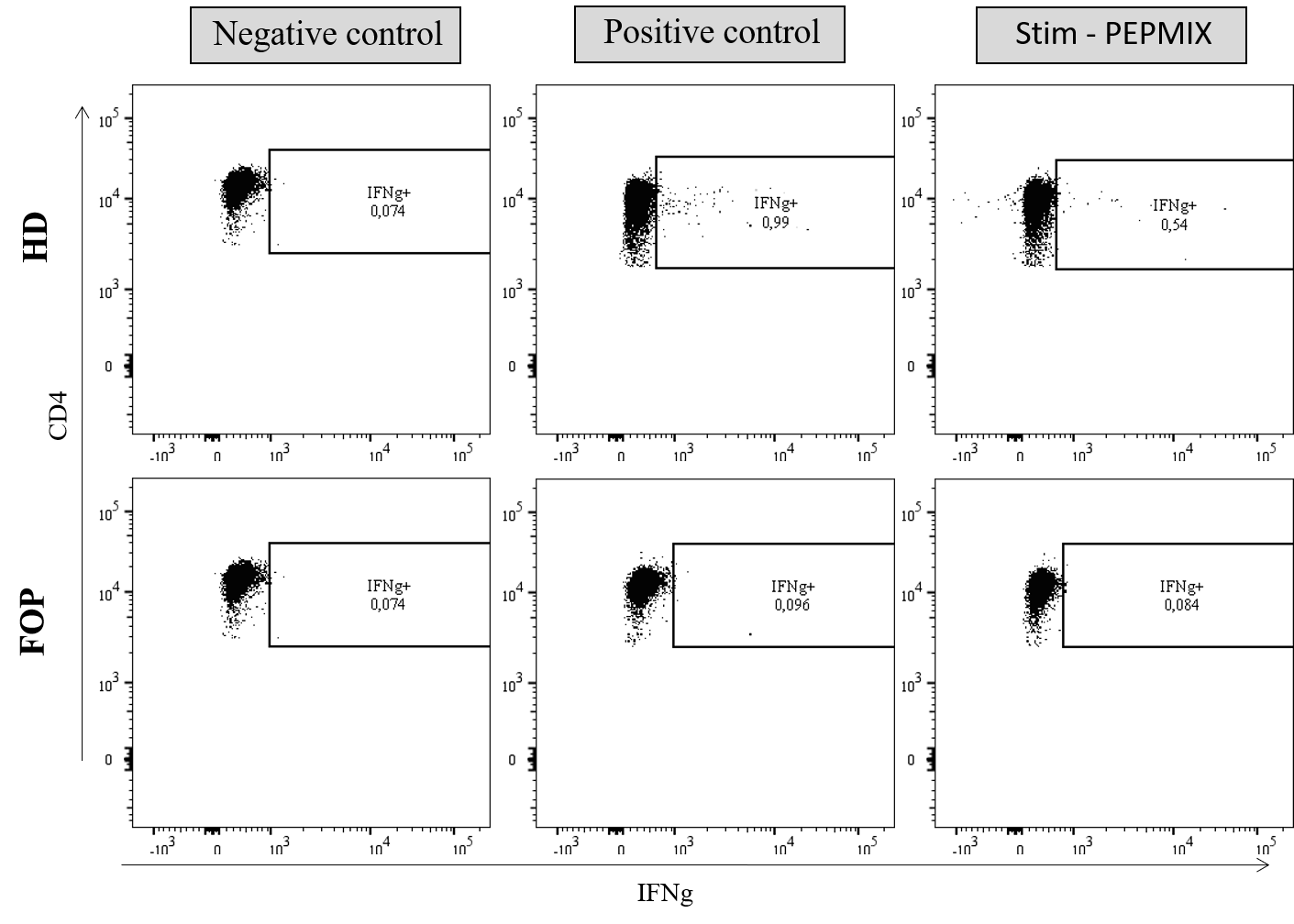

Supplement: Supplementary file 3 [file Image_3.tif]

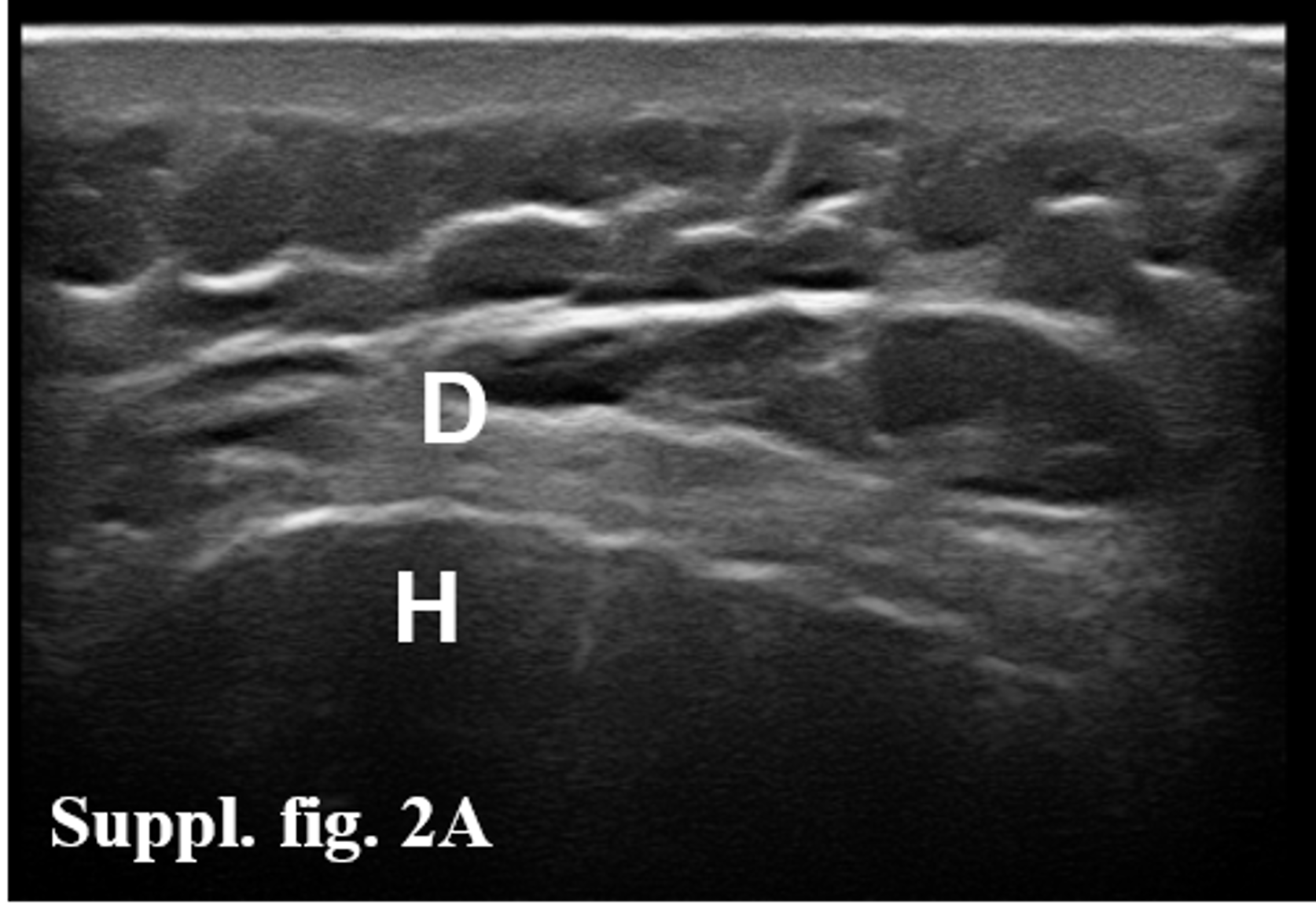

Supplement: Supplementary file 4 [file Image_4.tif]

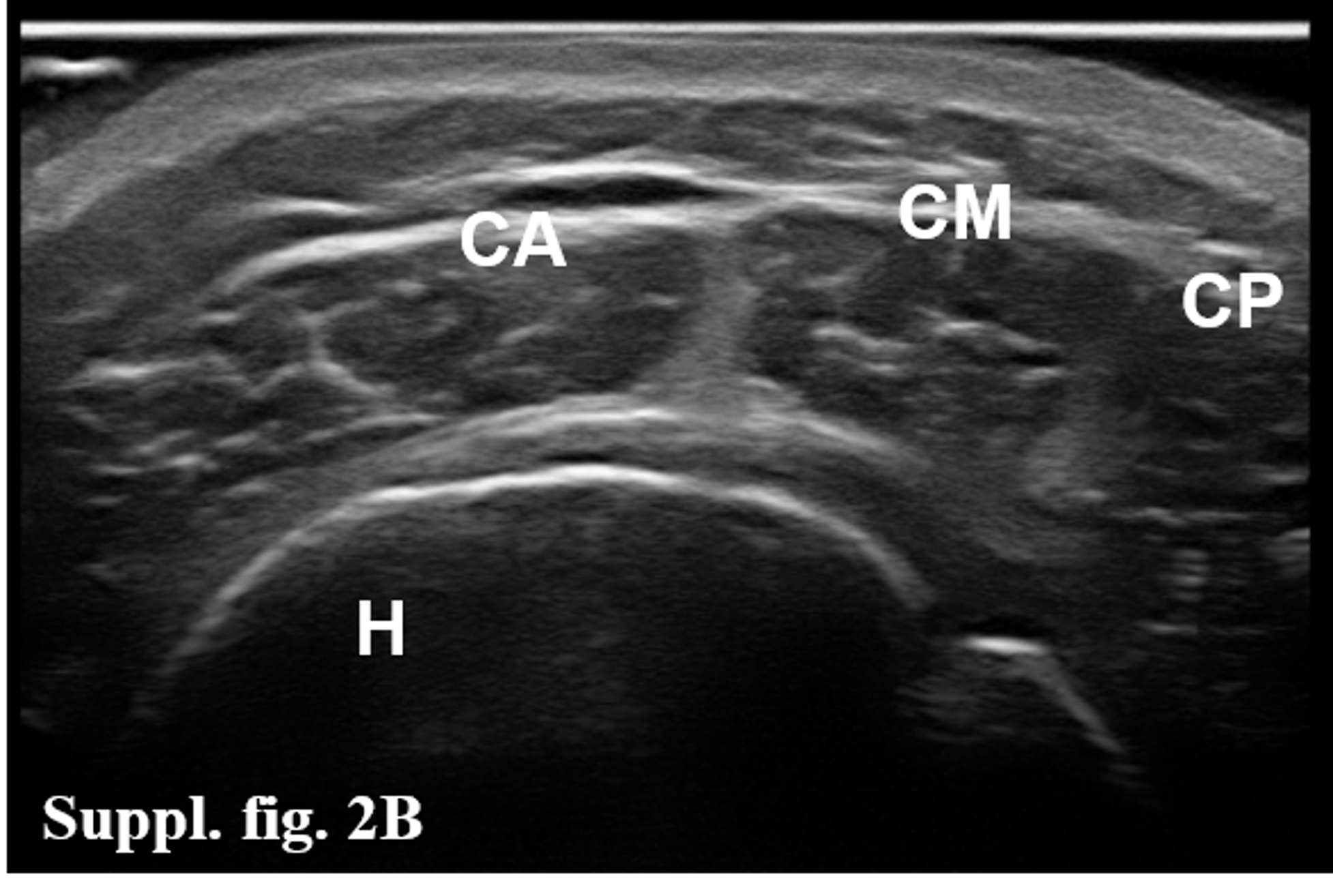

Supplement: Supplementary file 5 [file Image_5.tif]
