# Supplementary material for: SARS-CoV-2-specific humoral and cellular immune responses to BNT162b2 vaccine in Fibrodysplasia ossificans progressiva patients
Source: Front Immunol. 2022 Nov 9;13:1017232. doi: 10.3389/fimmu.2022.1017232 (PMC9682080; doi:10.3389/fimmu.2022.1017232)
Supplement: Supplementary file 6 [file Table_1.docx]

| **Antibody** | **Fluorochrom** | **Clone** | **Manufacturer** |
| --- | --- | --- | --- |
| Anti-Hu CD3 | Alexa Fluor 700 | MEM57 | Exbio, Vestec, Czech Republic |
| Anti-Hu CD4 | PE-Cy7 | MEM-241 | Exbio, Vestec, Czech Republic |
| Anti-Hu CD8 | PE-DyLight 594 | MEM-31 | Exbio, Vestec, Czech Republic |
| Anti-Hu TNFα | Brilliant Violet 421 | Mab11 | BioLegend, San Diego, CA, USA |
| Anti-IFN-γ | FITC | 4S.B3 | Exbio, Vestec, Czech Republic |

**Supplementary Table 1:** Characteristics of fluorescence activated cell sorting flurochrom conjugated monoclonal antibodies used for the assessment of specific T-cell (cellular) response.
