# Supplementary material for: SARS-CoV-2-specific humoral and cellular immune responses to BNT162b2 vaccine in Fibrodysplasia ossificans progressiva patients
Source: Front Immunol. 2022 Nov 9;13:1017232. doi: 10.3389/fimmu.2022.1017232 (PMC9682080; doi:10.3389/fimmu.2022.1017232)
Supplement: Supplementary file 7 [file Table_2.docx]

| **Parameter (unit)** | **Before (M)** | **After (M)** | **Before (F)** | **After (F)** | **Refferal values** |
| --- | --- | --- | --- | --- | --- |
| ***Hematology*** | | | | | |
| **LEU (E9/L)** | 6.2 | 6.2 | 6.2 | 4.6 | ***4.0 – 18.0*** |
| **RBC (E9/L)** | 5.61 | 5.35 | 4.18 | 4.02 | ***4.0 – 5.80*** |
| **HGB (g/L)** | 177 | 162 | 135 | 132 | ***135 – 175*** |
| **PLT (E9/L)** | 283 | 312 | 211 | 142 | ***150 – 400*** |
| **LYMPH (%)** | 0.216 | 0.239 | 0.33 | 0.351 | ***0.2 – 0.45*** |
| **MONO (%)** | 0.076 | 0.087 | 0.097 | 0.099 | ***0.02 – 12.0*** |
| **NEU (%)** | 0.694 | 0.656 | 0.557 | 0.553 | ***0.45 – 0.70*** |
| **EOS (%)** | 0.006 | 0.01 | 0.05 | 0.011 | ***0.0 – 0.05*** |
| **BAS (%)** | 0.005 | 0.006 | 0.003 | 0.004 | ***0.0 – 0.02*** |
| ***Biochemistry*** | | | | | |
| **ALP (ukat/L)** | 2.19 | 2.18 | 1.15 | 1.11 | ***0.66 – 2.2*** |
| **AST (ukat/L)** | 0.41 | 0.56 | 0.29 | 0.36 | ***0.16 – 0.72*** |
| **ALT (ukat/L)** | 0.46 | 0.49 | 0.22 | 0.29 | ***0.17 – 0.78*** |
| **GGT (ukat/L)** | 0.40 | 0.46 | 0.28 | 0.30 | ***0.14 – 0.84*** |
| **BILI-T (umol/L)** | 18.1 | 19.4 | 12.8 | 13.6 | ***0.41 – 3.24*** |
| **UREA (mmol/L)** | 3.8 | 3.3 | 3.8 | 3.9 | ***5.0 – 21.0*** |
| **CREAT (umol/L)** | 37 | 39 | 55 | 55 | ***55 - 96*** |
| **hs-CRP (mg/L)** | 5.9 | 6.6 | <0.5 | <0.5 | ***0.0 – 5.0*** |
| ***Immunology*** | | | | | |
| **IgG (g/L)** | 14.7 | 13.3 | 12.4 | 12.4 | ***7.65 – 13.6*** |
| **IgA (g/L)** | 5.07 | 4.69 | 2.07 | 2.02 | ***0.91 – 2.9*** |
| **IgM (g/L)** | 1.71 | 1.62 | 2.31 | 2.25 | ***0.47 – 1.95*** |
| **C3 (g/L)** | 1.71 | 1.62 | 0.85 | 0.74 | ***0.85 – 2.25*** |
| **C4 (g/L)** | 0.23 | 0.21 | 0.24 | 0.23 | ***0.14 – 0.35*** |
| **RF-IgG (IU/mL)** | 4.2 | 5.2 | 3.8 | 4.6 | ***0.0 – 20.0*** |
| **RF-IgA (IU/mL)** | 2.1 | 2.2 | 1.0 | 2.1 | ***0.0 – 20.0*** |
| **RF-IgM (IU/mL)** | 0.8 | 1.6 | 2.3 | 8.6 | ***0.0 – 20.0*** |
| **ANA (IF)** | negative | weakly positive (1:80) | negative | negative | ***negative/positive*** |
| **ANCA (IF)** | negative | negative | negative | negative | ***negative/positive*** |

**Supplementary Table 2:** Laboratory parameters before and 3 months after vaccination in male (M) and female (F) patient with Fibrodysplasia ossificans progressiva (LEU, leukocytes; RBC, red blood count; HGB, hemoglobin; PLT, platelets; LYMPH, lymphocytes; MONO, monocytes NEU, neutrophils; EOS, eosinophils; BAS, basophils; ALP, alkaline phosphatase; AST, aspartate aminotransferase; ALT, alanine aminotransferase; GGT, gamma glutamyl transferase; BILI-T, total bilirubin; CREAT, creatinine; Ig, immunoglobulin; C3, complement component 3; C4, complement component 4; RF, rheumatoid factor; ANA, anti-nuclear autoantibodies; ANCA, anti-neutrophil cytoplasmic antibodies
